# Retinal Microvascular Abnormalities in Neurofibromatosis Type 1 Associated with Congenital Retinal Macrovessels

**DOI:** 10.1155/2013/604191

**Published:** 2013-05-23

**Authors:** Shinji Makino, Katsuhisa Endoh, Hironobu Tampo

**Affiliations:** ^1^Department of Ophthalmology, Jichi Medical University, Tochigi, 3311-1 Yakushiji, Shimotsuke 329-0498, Japan; ^2^Department of Ophthalmology, Hokuto City Shiokawa Hospital, Yamanashi, 773 Fujita, Sudama, Hokuto 408-0114, Japan

## Abstract

Here, we report a case of retinal microvascular abnormalities in a patient with neurofibromatosis type 1 (NF1) associated with congenital retinal macrovessels. An abnormal retinal macrovessel, crossing the macula horizontally, was detected in the right eye. Additionally, retinal microvascular abnormalities were detected. Eight years after the initial visit, the retinal microvascular abnormalities were noted to have changed substantially. We speculate that retinal microvascular abnormalities in NF1 may change dynamically over the years.

## 1. Introduction

The presence of congenital retinal macrovessels, a phenomenon first described by Brown et al. [1] in 1982, is rare. In patients with this condition, a vein in 1 eye is seen to cross the horizontal raphe and the macula. The associated visual disturbance is mild, and such cases may be detected only incidentally [1–5].

Seemingly unrelatedly, neurofibromatosis type 1 (NF1), an autosomal dominant disorder with a high mutation rate, is considered a neurocristopathy characterized by pathological hamartomatous proliferations of neural crest-derived tissues. A minimum of 2 of the following criteria are required for diagnosis: 6 or more café-au-lait spots, 2 or more cutaneous neurofibromas, 1 or more plexiform neurofibromas, axillary or groinal freckling, optic glioma, 2 or more iris Lisch nodules, distinctive bony lesions, and a first-degree relative with NF1 [6]. Among these criteria, iris Lisch nodules are frequently observed and well recognized. However, retinal and choroidal lesions have been considered unusual in eyes with this disease. Few reports have described microvascular retinal abnormalities in patients with NF1. These typically present a corkscrew configuration [7–10]. 

Herein, we report a case of retinal microvascular abnormalities in NF1 associated with the presence of a congenital retinal macrovessel. The angiographic aspect of this case was described previously [11]. Interestingly, this patient's retinal microvascular abnormalities changed dynamically throughout the follow-up period. 

## 2. Case Report 

**Figure 1:**
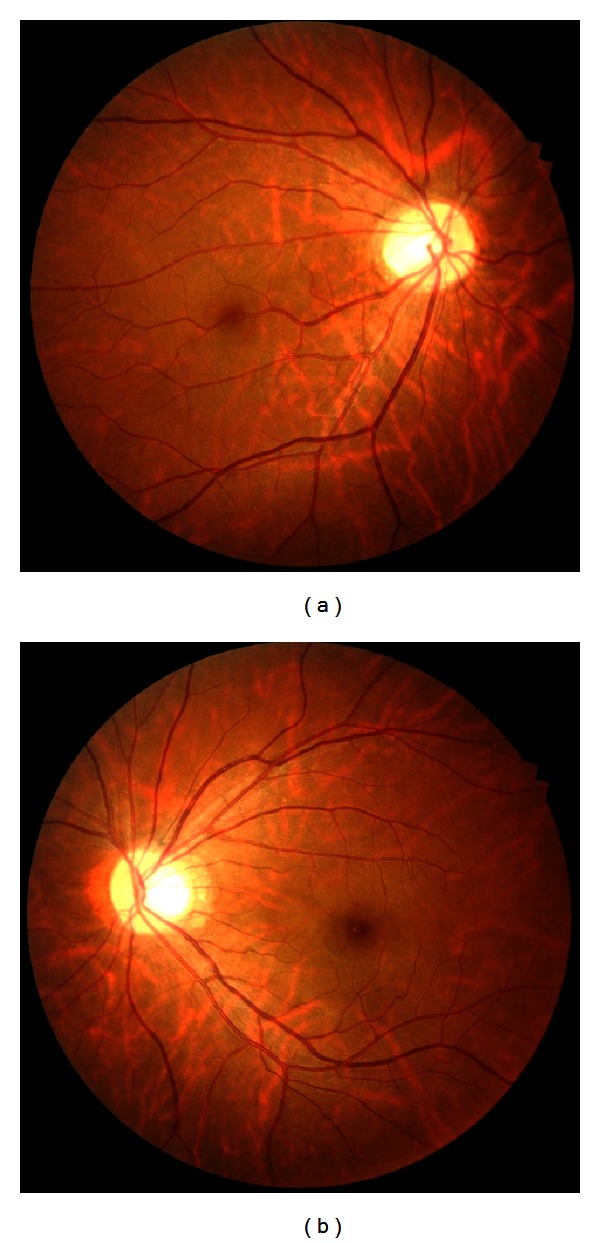
Right (a) and left (b) fundus photographs. Note a congenital retinal macrovessel crossing the macula horizontally in the right eye.

**Figure 2:**
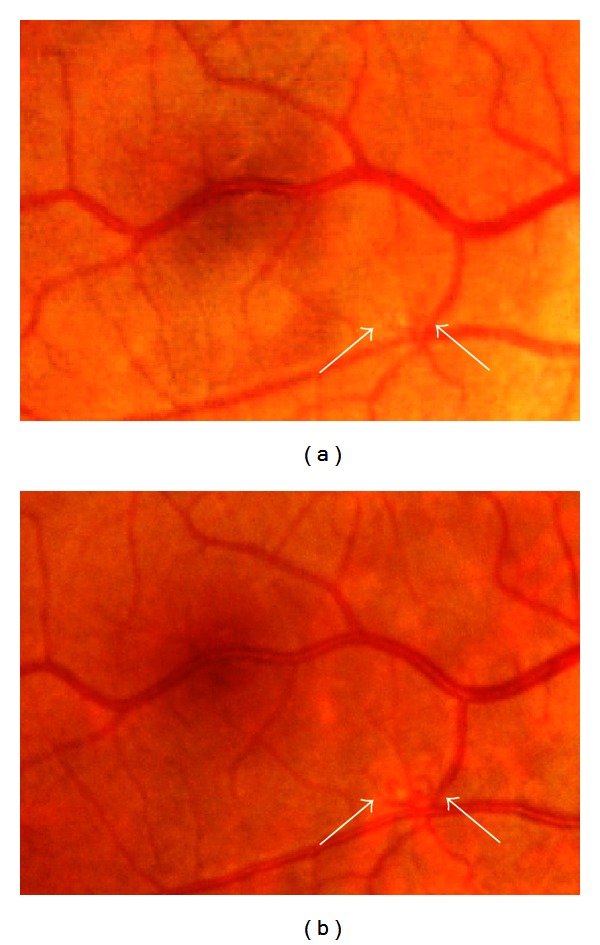
High-magnification images of the right fundus. Note dynamic changes to the microvascular abnormality (arrow) (b) in comparison to its appearance at the initial visit (a).

**Figure 3:**
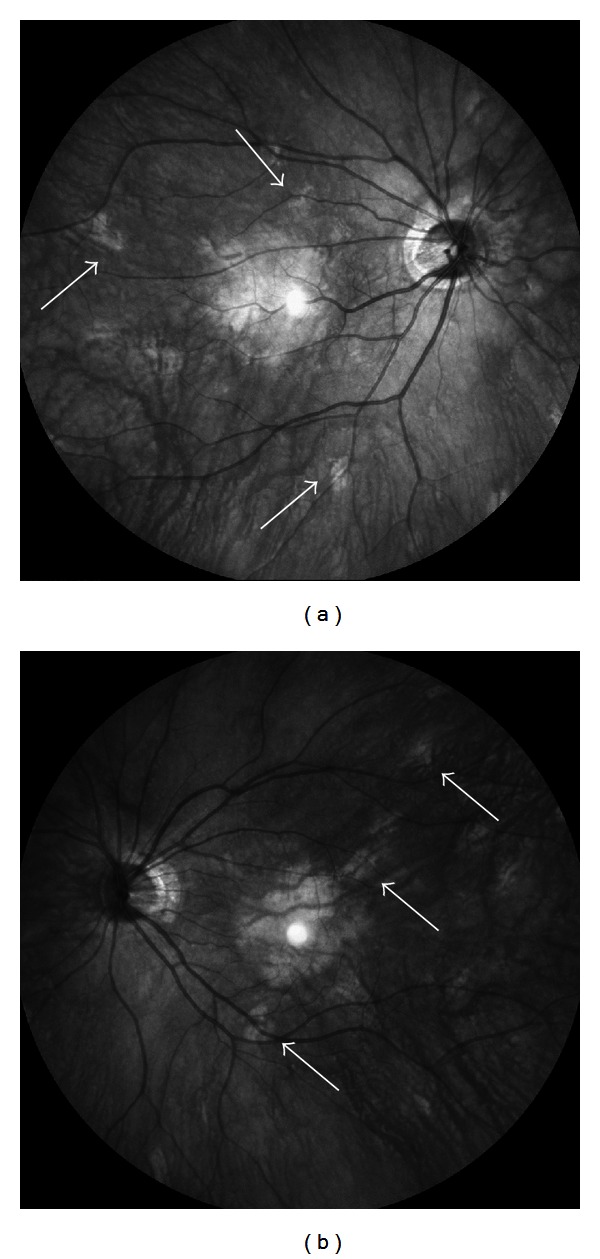
Right (a) and left (b) infrared fundus autofluorescence images. Note multiple bright, patchy lesions in the posterior poles of both eyes. The hyperreflective point at the center of the image is an optical artifact.

A 30-year-old woman with NF1 was referred to our clinic for an ophthalmological examination. The NF1 diagnosis was made on the basis of numerous café-au-lait spots and cutaneous neurofibromas. Her mother had also been diagnosed with NF1. Her personal medical history added no significant information. The patient had been aware of reduced visual acuity in her right eye since childhood. Her best-corrected visual acuity was 0.8 and 1.2 in the right and left eye, respectively. There were at least 3 Lisch nodules on each iris. Ophthalmoscopic examinations of the fundi did not reveal any abnormalities in the left eye. In the right eye, an abnormal macrovessel, crossing the macula horizontally, was detected (Figures 1(a) and 1(b)). Retinal microvascular abnormalities were detected as well (Figure 2(a)). The patient was followed up for eight years, during which time the retinal microvascular abnormalities in the right eye exhibited dynamic changes (Figure 2(b)). The patient's fundus was examined using infrared fundus autofluorescence (IR-FAF) (Heidelberg Retina Angiograph 2, Heidelberg Engineering, Heidelberg, Germany). IR-FAF revealed multiple bright, patchy lesions in the posterior pole of the choroid (Figures 3(a) and 3(b)).

## 3. Discussion

This is the first reported case of retinal microvascular abnormalities that change dynamically over time in a patient with NF1 associated with a congenital retinal macrovessel.

Congenital retinal macrovessels are large aberrant retinal vessels that cross the horizontal raphe. They are predominantly unilateral, typically benign, and typically are not associated with visual deficits. The condition is usually asymptomatic, and there are no visual complaints. A reduction of vision in such cases is rare. When it has occurred, it has been attributed to Valsalva retinopathy, the rupture of capillary alterations, foveal cysts, or the mere presence of an abnormal vessel in the foveal area [1–5].

In 2002, Muci-Mendoza et al. [7] described a novel retinal finding in NF1: distinctive microvascular abnormalities were noted in 12 of 32 (37.5%) patients. In 10 cases, the anomaly was very subtle, involving a second- or third-order venule, a tributary of the superior or inferior temporal veins, or, less frequently, the nasal veins. The tortuous vessel had a corkscrew appearance and ended in a minute tuft. In two of the patients, however, more striking abnormalities were observed: a venovenous anastomosis in the nasal retina and an extensive arteriovenous malformation coexisting with an epiretinal membrane. Two of these 12 patients also had choroidal neurofibromas. Fluorescein angiography was performed in each case, but failed to reveal any leakage in 50% of those examined. None of the patients reported any vision disturbances. Such vascular abnormalities may therefore represent a new retinal marker for NF1. Karadimas et al. [8] also described an NF1 patient with retinal microvascular abnormalities. Muci-Mendoza et al. [7] believe that these retinal microvascular abnormalities are congenital and stable, as evidenced by their unchanging appearance over the years. However, the microvascular abnormalities observed in our case exhibited dynamic changes over the years. We speculate that this atypical presentation is likely associated with the presence of NF1 as a comorbid condition.

In our case, IR-FAF revealed multiple bright, patchy lesions. Yasunari et al. [12] suggested that choroidal abnormalities occurred more frequently than Lisch nodules in the iris. They also suggested that bright, patchy choroidal lesions should be a new diagnostic criterion for NF1. In a recent study with a large number of NF1 patients, Viola et al. [13] reported that IR-FAF imaging revealed choroidal nodules in 82% of NF1 patients as compared with 7% of healthy patients. The extent of choroidal involvement is likely to vary among patients and increases with age [14]. Long-term follow up and additional cases are necessary to further characterize the changing appearance of retinal microvascular abnormalities in NF1 patients.
